# Propionic acid in multiple sclerosis: a phase 2b, double-blind, randomized placebo-controlled trial

**DOI:** 10.1093/brain/awag099

**Published:** 2026-07-06

**Authors:** Tobias Moser, Wolfgang Hitzl, Tiago Lerda-Casaccia, Michael Unterhofer, Rina Demjaha, Maria Martinez-Serrat, Michael Khalil, Andrea Harrer, Belinda Böhm, Peter Hofbauer, Janne Cadamuro, Ursula Huber-Schönauer, Eugen Trinka, Peter Wipfler

**Affiliations:** Department of Neurology, Christian Doppler University Hospital, Paracelsus Medical University and Center for Cognitive Neuroscience, European Reference Network EpiCARE, Salzburg 5020, Austria; Department of Ophthalmology and Optometry, Paracelsus Medical University Salzburg, Salzburg 5020, Austria; Research Program Experimental Ophthalmology & Glaucoma Research, Paracelsus Medical University Salzburg, Salzburg 5020, Austria; Department of Neurology, Christian Doppler University Hospital, Paracelsus Medical University and Center for Cognitive Neuroscience, European Reference Network EpiCARE, Salzburg 5020, Austria; Department of Neurology, Christian Doppler University Hospital, Paracelsus Medical University and Center for Cognitive Neuroscience, European Reference Network EpiCARE, Salzburg 5020, Austria; Department of Neurology, Medical University of Graz, Graz 8036, Austria; Department of Neurology, Medical University of Graz, Graz 8036, Austria; Department of Neurology, Medical University of Graz, Graz 8036, Austria; Department of Neurology, Christian Doppler University Hospital, Paracelsus Medical University and Center for Cognitive Neuroscience, European Reference Network EpiCARE, Salzburg 5020, Austria; Department of Dermatology and Allergology, University Hospital Salzburg, Paracelsus Medical University, Salzburg 5020, Austria; Department for Health Sciences, Physiotherapy, Salzburg University of Applied Sciences, Puch/Salzburg 5412, Austria; Landesapotheke Salzburg, Betrieb des Landes Salzburg, Salzburg 5020, Austria; Department of Laboratory Medicine, Paracelsus Medical University, Salzburg 5020, Austria; Department of Nuclear Medicine and Endocrinology, University Hospital Salzburg, Paracelsus Medical University, Salzburg 5020, Austria; Department of Neurology, Christian Doppler University Hospital, Paracelsus Medical University and Center for Cognitive Neuroscience, European Reference Network EpiCARE, Salzburg 5020, Austria; Neuroscience Institute, Christian Doppler University Hospital, Paracelsus Medical University and Center for Cognitive Neuroscience, Salzburg 5020, Austria; Department of Neurology, Christian Doppler University Hospital, Paracelsus Medical University and Center for Cognitive Neuroscience, European Reference Network EpiCARE, Salzburg 5020, Austria

**Keywords:** multiple sclerosis, propionic acid, neurofilament light chain, add-on therapy, neuroprotection, clinical trial

## Abstract

Propionic acid (PA), a microbial-derived short-chain fatty acid, contributes to intestinal barrier integrity, systemic immune regulation, and neuronal function. Individuals with multiple sclerosis show reduced PA levels, and open-label data have suggested beneficial immunomodulatory and clinical effects of supplementation.

The Multiple sclerosis And DisAbility Improvement (MADAI) trial was a randomized, double-blind, placebo-controlled, single-centre, phase 2b study designed to evaluate the efficacy and safety of PA as an add-on therapy in adults with clinically stable multiple sclerosis. Between April 5 and 29 May 2024, 101 adults (64% women; mean age 45 years) were randomly assigned in a 2:1 ratio to receive PA 500 mg twice daily or matching placebo for 90 days. The primary outcome was the change in serum neurofilament light chain (sNfL) concentration, a biomarker of neuroaxonal damage, adjusted for age, body mass index, creatinine, and baseline sNfL. Secondary outcomes included physical and cognitive performance measures and patient-reported outcomes, including fatigue and quality of life scores.

sNfL levels were significantly reduced in the PA group {−17.9%; from 9.77 pg/ml [95% confidence interval (CI) 9.00 to 10.60] to 8.02 pg/ml (95% CI 7.36 to 8.73); mean difference 1.75 pg/ml (95% CI 0.9 to 2.6); *P* = 0.000025}, while no significant change was observed in the placebo group. The adjusted mean difference in sNfL levels between the PA and placebo groups at follow-up was 0.91 pg/ml (95% CI 0.02 to 1.79; *P* = 0.045). Reductions in sNfL were also observed among participants in the PA arm receiving moderate-to-high efficacy disease-modifying therapies (*n* = 41; *P* = 0.0001), including those on anti-CD20 treatment (*n* = 27; *P* = 0.0005). There was a trend towards improvement in motor fatigue in the PA group. No serious adverse events related to the study medication occurred.

PA supplementation was well tolerated and associated with significant reductions in sNfL, suggesting attenuation of neuroaxonal injury in multiple sclerosis. These findings support further evaluation of PA as an add-on treatment in larger, long-term studies.

## Teaser

Moser *et al.* evaluated the efficacy and safety of propionic acid (PA) as an add-on therapy in adults with clinically stable multiple sclerosis. They found that PA supplementation was well-tolerated and was associated with significant reductions in serum neurofilament light chain levels and a trend toward improvement in fatigue.

**See Sandgren *et al.* (https://doi.org/10.1093/brain/awag192) for a scientific commentary on this article.**

## Introduction

Multiple sclerosis is a chronic disorder of the CNS, characterized by inflammatory and neurodegenerative mechanisms contributing to accumulation of disability.^1^ The disease primarily affects young adults of working age, resulting in a substantial personal and socioeconomic burden.^2,3^ While current disease-modifying therapies (DMTs) effectively control inflammatory activity, including clinical relapses and focal lesion formation on MRI, they do not primarily act within the CNS itself.^4^ Despite excellent reductions in relapse rates, a considerable proportion of individuals receiving ocrelizumab or natalizumab exhibit progression independent of relapse activity (PIRA), underscoring ongoing disability accumulation unrelated to overt inflammatory activity.^5,6^ Thus, preventing long-term disability and promoting sustained neurological improvement remain unmet needs in the treatment of multiple sclerosis across all disease stages.

The rising incidence of multiple sclerosis has been linked to environmental factors, including Western dietary patterns characterized by high fat and salt intake and low fibre consumption.^7^ Emerging evidence indicates that the intestinal microbiome in individuals with multiple sclerosis differs from that of healthy controls and that the gut-brain axis influences the disease course.^8^ Despite growing insights into the complex interplay between the gut, brain, and immune system, there are currently no established dietary recommendations for individuals with multiple sclerosis.

Propionic acid (PA) is a short-chain fatty acid (SCFA) produced by the microbial fermentation of dietary fibres and indigestible carbohydrates. Following intestinal absorption and entry into systemic circulation, PA is capable of crossing the blood–brain barrier and reaching the CNS.^9,10^ Importantly, PA levels are reduced in both the stool and blood of individuals with multiple sclerosis.^10-12^ Moreover, an open-label multiple sclerosis study reported that, once supplemented, PA had beneficial effects on relapse rates, disability progression and brain atrophy.^10^

The Multiple sclerosis And DisAbility Improvement (MADAI) trial was designed to evaluate the efficacy and safety of supplementary PA in multiple sclerosis as an add-on therapy in a prospective, randomized, placebo-controlled setting. The primary objective was to assess PA-induced changes in serum neurofilament light chain (NfL), a biomarker for acute and chronic neuroaxonal injury.^13^ Reductions in plasma or serum NfL (sNfL) have been shown to parallel the efficacy of DMTs, supporting its use as a sensitive marker of treatment response in phase 2 and as a secondary outcome in phase 3 clinical trials.^14-16^

## Materials and methods

### Study design

We conducted a prospective, double-blind, randomized, sample size planned, placebo-controlled, single-centre study at the multiple sclerosis outpatient clinic of the Christian Doppler University Hospital Salzburg, Austria. The study followed CONSORT guidelines. Recruitment began in Q1 2024, and participants were enrolled by Q2 2024. The active study duration was 90 days, with follow-up visits in Q3 2024. We extracted demographics and data regarding multiple sclerosis history from patients’ source records.

The study was conducted in accordance with the Declaration of Helsinki. Ethical approval was obtained from the ethics committee of the Federal State of Salzburg (Ethikkommission Land Salzburg; approval number 1026/2024), and all participants provided written informed consent before trial-related procedures commenced. The MADAI trial was registered at ClinicalTrials.gov (identifier: NCT06402487), and its status is completed.

### Participants

**Figure 1:**
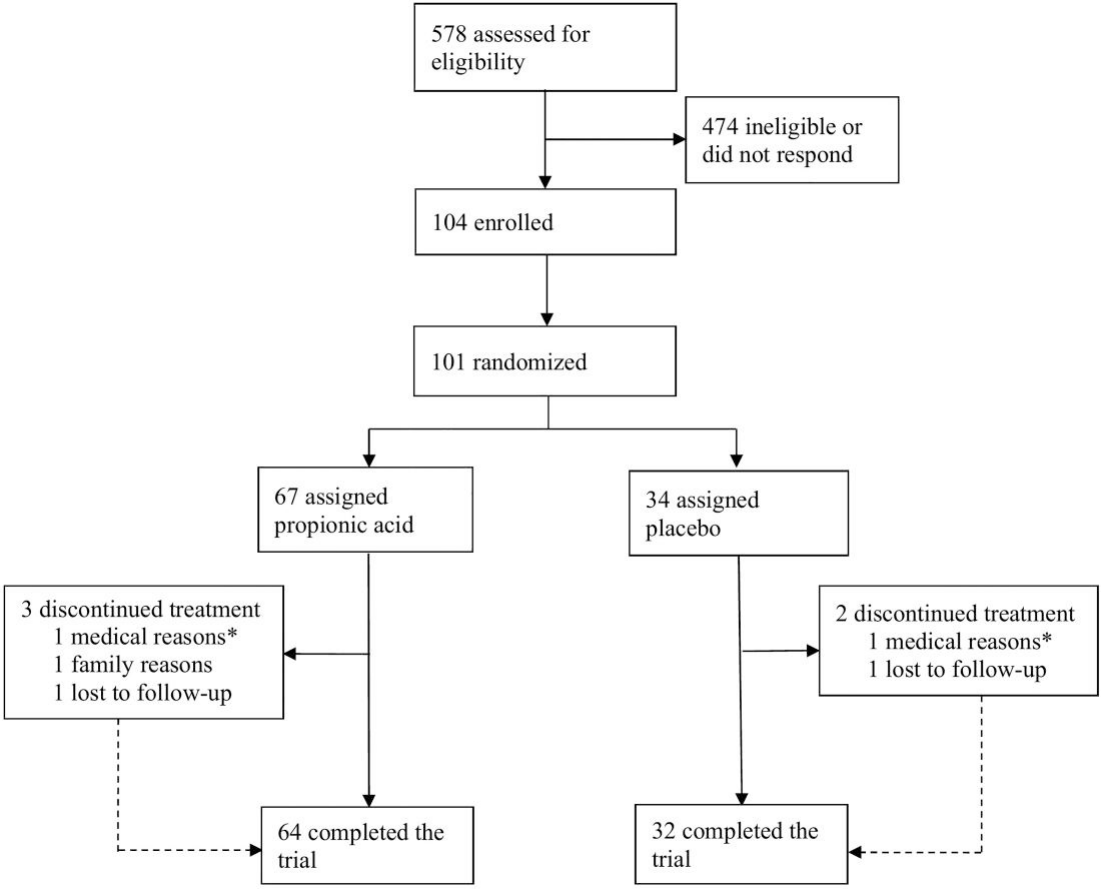
**Trial profile**. *Withdrawals were considered unrelated to the trial.

A total of 578 individuals from the local multiple sclerosis registry of the Christian Doppler University Hospital, Salzburg, Austria, were contacted via letters and invited to participate in the study (Fig. 1). Eligible participants were adults aged 18–70 years with a diagnosis of clinically stable multiple sclerosis (no relapse in the preceding three months), established according to the 2017 McDonald criteria. Inclusion required the presence of either oligoclonal bands (OCBs) or a positive intrathecal measles–rubella–zoster (MRZ) reaction. All participants provided written informed consent and were required to have available blood samples at both baseline and study completion. In addition, women of childbearing age were required to have a negative pregnancy test. Exclusion criteria were an existing supplementation with PA, a positive JC virus titre under natalizumab therapy, and the presence of severe ongoing systemic diseases. Acute structural neurological conditions, such as brain tumours or stroke, also led to exclusion. Sex assigned at birth was self-reported by participants at study enrolment using predefined categories (male, female, or other). Where relevant, analyses stratified by sex refer to this self-reported designation.

### Randomization and masking

Eligible participants were randomly allocated in a 2:1 ratio to receive either PA 500 mg twice daily or a matching placebo for a planned duration of 90 days. The selected dose of 500 mg PA twice daily was based on the regimen used in a previous open-label study demonstrating immunomodulatory and clinical effects in individuals with multiple sclerosis.^10^ Randomization was carried out using computer-generated numbers with block randomization, employing variable block sizes of four, eight and twelve. All computations were conducted using PASS 2022 (Power Analysis and Sample Size Software, Version 22; NCSS, LLC, Kaysville, Utah, USA). The randomization list was password-protected and could not be modified. The study medication (verum and placebo) was packaged in identical, consecutively numbered containers. To maintain the double-blind design, only designated pharmacy staff and the statistician were informed of the group assignments. Participants, investigators, and outcome assessors were unaware of treatment assignments and efficacy outcomes throughout the trial and had no access to randomization codes.

### Procedures

PA or placebo were recommended to be administered with food in a 1-0-1 schedule; depending on food intake, an alternative dosing of 0-1-1 was also permitted to minimize (gastrointestinal) side effects. Sodium propionate 500 mg was provided by ZeinPharma. The product has legal market authorization confirmed by a certificate of marketability issued by Chemical Laboratory Dr Mang (Frankfurt am Main, Germany), allowing its use in human clinical studies. The pharmacy at the University Hospital Salzburg manufactured the placebo capsules and was responsible for final packaging and labelling of the study medications.

The study medication was administered as an add-on approach, and therefore, participation in this trial had no impact on concurrent DMTs. For subgroup analyses, participants were categorized based on the use of concomitant DMTs, particularly anti-CD20 therapies. The following treatments were classified as moderate-to-high efficacy DMTs: anti-CD20 therapies, sphingosine-1-phosphate (S1P) receptor modulators, cladribine (within four years of treatment initiation), alemtuzumab (within four years of treatment initiation), and natalizumab. Moreover, we recorded whether participants initiated a new DMT within 12 months of baseline to account for a potential confounding effect of DMT initiation/escalation on sNfL.

Study medication was dispensed at the baseline visit along with a structured dosing schedule. All participants received a side effect diary, which they were asked to complete throughout the study and return at the end-of-trial visit. All assessments were conducted at study start (baseline) and after 90 days of PA or placebo intake (follow-up).

For sNfL assessment, serum samples obtained at both timepoints were centrifuged (3000*g*, 10 min), and serum aliquots were stored at −80°C until shipment on dry ice after study completion. sNfL concentrations were measured on Simoa^®^ HD-X analyser (Quanterix^®^) at the Medical University of Graz, Austria. Analyses were performed in accordance with the manufacturer’s instructions. All samples were measured in duplicate using the same kit lot to ensure consistency. The measurements were conducted by laboratory personnel blinded to sample identities and clinical information. All sample measurements, including quality controls, showed intra-assay coefficients of variation below 20%, in line with established analytical performance standards.

Blood tests with immediate analysis were performed at both study visits and included complete blood count, C-reactive protein (CRP), renal [creatinine, estimated glomerular renal function (eGFR)] and liver function panels [aspartate transaminase (AST), alanine transaminase (ALT), alkaline phosphatase (ALP), gamma-glutamyltransferase (GGT)], electrolytes (sodium, potassium and chloride). Pregnancy was excluded at baseline. Cognitive performance was assessed using the Symbol Digit Modalities Test (SDMT), which measures processing speed by having participants match symbols to numbers within 90 s. Physical function was evaluated with the Nine-Hole Peg Test (9HPT) for manual dexterity and the 10-Meter Walk Test (10mWT) for walking speed. Quality of life was measured using the 36-Item Short Form Health Survey questionnaire (SF-36), providing scores for eight domains and two summary components [Physical Component Summary (PCS) and Mental Component Summary (MCS)], where higher scores reflect better health status. Fatigue was assessed with the Fatigue Scale for Motor and Cognitive Functions (FSMC), distinguishing motor (FSMCmot) and cognitive (FSMCcog) fatigue, while daytime sleepiness was measured using the Epworth Sleepiness Scale (ESS). All questionnaires were administered online at baseline and follow-up, and responses were collected electronically for analysis. A detailed explanation of the procedures used is given in the Supplementary material, ‘Methods’ section. Cerebral MRI (cMRI) data were retrieved from patient records. We included assessments covering baseline and three months prior to study start to evaluate radiological disease activity, defined by the presence of new T2 lesions or gadolinium-enhancing lesions, compared with the most recent previous MRI scan.

### Outcomes

The primary endpoint was the reduction in sNfL levels from baseline to the end of the trial. Secondary outcomes included changes in cognitive and physical performance, as measured by the SDMT score, the 9HPT (the fastest time recorded across all attempts in seconds), and the 10mWT (in seconds), as well as changes in patient-reported outcomes on quality of life (PCS and MCS of SF-36), fatigue [assessed separately for motor (FSMCmot) and cognitive (FSMCcog) domains using the FSMC and the overall score FSMCtot] and sleepiness (ESS). Safety was assessed via documentation of treatment-emergent adverse events and included laboratory assays. Side effects were recorded at any time after the study medication was provided.

### Statistical analysis

All statistical computations, including sample size calculation, randomization and analysis, were performed by a professional statistician (W.H.). Sample size computation was based on an estimated standard deviation of 2.0 pg/ml for sNfL levels, derived from a study investigating dietary interventions in multiple sclerosis,^17^ assuming normal distributions, variance homogeneity, a significance level of 5%, a drop-out rate of 5% in each group and a group allocation ratio of 2:1. With group sample sizes of *n*_1_ = 57 (verum) and *n*_2_ = 28 (placebo), the study had 90% power to detect a between-group difference of 1.36 pg/ml using a one-sided two-sample equal-variance *t*-test. This target difference corresponds to a ≥10% reduction in sNfL over 90 days, based on an average baseline sNfL of ∼13.6 pg/ml, calculated as the unweighted mean of published baseline values from the OPERA I/II relapsing multiple sclerosis cohorts (10.7 and 10.4 pg/ml) and the early multiple sclerosis cohort reported by Lin *et al*.^18,19^ (19.7 pg/ml). This reduction was considered biologically meaningful and achievable within a feasible sample size. After adjusting for potential dropouts, the final planned sample sizes were *n*_1_ = 60 and *n*_2_ = 30. Outcome and safety analyses were conducted on the intention-to-treat population.

Categorical data were analysed using Pearson’s chi-squared and Fisher’s Exact test. Continuous variables (besides EDSS) were tested for normality using the Kolmogorov-Smirnov test, and generalized estimation equation models (GEE) with corresponding least significant differences were used to compare means. For sNfL, GEE models were based on Tweedie distribution (Supplementary Figs 1 and 2) with logarithm as the link function. Treatment effect was estimated as the interaction between visit and treatment arm according to the randomization, with sNfL as the dependent variable. Age, body mass index (BMI), creatinine and sNfL at baseline were used as continuously distributed covariates to compute adjusted means (Supplementary Table 1). Detailed parameter estimates for all outcomes, including coefficients, standard errors and covariate values, as well as planned contrasts of adjusted mean values, are provided in Supplementary Tables 2–6. The primary objective of the study was to assess reductions in sNfL, and the corresponding statistical tests were one-sided. Secondary end points were analysed using analogous GEE models. In cases of missing baseline data, case-wise deletion was applied. For missing data at follow-up, no case-wise deletion was performed, as GEE models can accommodate missing values by estimating an internal covariance matrix. However, for the FSMC and the SF-36, missing item responses were addressed using a person-mean imputation method. If fewer than 20% of the items within a given scale were missing for a participant, the mean of their available responses on that scale was calculated and used to replace the missing values. Following imputation, total scores for the FSMC were recalculated accordingly. For EDSS scores, the Mann–Whitney U-test was used, and descriptive values were reported as median with 25th and 75th quartiles; otherwise, mean and standard deviation were used.

Tests for changes in secondary outcomes were two-sided. As these analyses were exploratory in nature, multiplicity adjustments were not applied. Whisker plots with 95% confidence intervals (CI) for means are provided to illustrate the results. *P*-values smaller than 5% were considered statistically significant. All results were computed using Cloud Software Group, Inc. (2023), PASS (2022) and IBM SPSS Statistics (Version 29).

## Results

Between 5 April 2024 and 29 May 2024, a total of 101 adults (64% women, mean 45 years) underwent randomization (Fig. 1): 67 were assigned to PA and 34 to placebo. The study was completed by 96 (95%) individuals. One participant (placebo arm) had a clinical relapse within 1 week of baseline and another (verum arm) had lumbar spine surgery within 2 weeks of the follow-up; the respective sNfL values were excluded from the primary analysis. The number of available data for secondary outcomes is summarized in Supplementary Table 7.

Demographic and disease-related characteristics of the study population were comparable between the two treatment groups (Table 1). Cerebral MRI data covering the recruitment and baseline period were available for 51 participants (76%) in the PA group and 26 participants (76%) in the placebo group. Compared with their most recent prior MRI scans, four participants (15%) in the placebo group had new T2 lesions, including one (4%) with gadolinium enhancement, whereas only one participant (2%) in the PA group had a new T2 lesion without gadolinium enhancement.

**Table 1:** **Demographics and baseline characteristics of the study population** (***n* = 101**)

	PA (*n* = 67)	Placebo (*n* = 34)	*P*
Female	46 (69%)	19 (56%)	0.27
Age, years	45.3 (12.4)	44.7 (11.2)	0.82
BMI	26.0 (7.2)	25.8 (5.7)	0.90
Time since MS diagnosis, years	10.4 (8.8)	13.0 (8.5)	0.16
sNfL; mean, pg/ml^a^	9.8 (9.0–10.6)	9.9 (8.9 to 11.0)	0.81
Vitamin D, ng/ml	41.1 (28.7)	39.9 (27.6)	0.84
10mWT; time, s	4.0 (2.6)	5.0 (4.3)	0.16
9-HPT; time, s	16.0 (6.3)	17.7 (9.0)	0.26
SDMT, mean	51.3 (17.0)	51.7 (13.5)	0.90
EDSS, median	1.5 (1.0–3.5)	1.0 (0–4.0)	0.84
Smokers	15 (23%)	8 (24%)	0.93
MS type	–	–	0.55
RRMS	52 (78%)	27 (79%)	–
SPMS	7 (10%)	5 (15%)	–
PPMS	8 (12%)	2 (6%)	–
Moderate-to-high efficacy DMTs	41 (61%)	18 (53%)	0.52
Months on moderate-to-high efficacy DMTs, mean	32 (23–41)	24 (14–35)	0.33
Anti-CD20 therapies	27 (40%)	12 (36%)	0.82
DMTs start/switch within 12 months from baseline			
Yes	9 (13%)	8 (24%)	0.26
No	58 (87%)	26 (76%)	0.26
Anti-CD20 start/switch within 12 months from baseline	9 (13%)	7 (21%)	0.39

Data are presented as *n* (%), median (IQR), mean (SD) or mean (CI). 10mWT = 10-Meter Walk Test; 9-HPT = 9-Hole Peg Test; BMI = body mass index; CI = 95% confidence interval; DMTs = disease-modifying therapies; EDSS = Expanded Disability Status Scale; IQR = interquartile range; MS = multiple sclerosis; PA = propionic acid; PPMS = primary progressive multiple sclerosis; RRMS = relapsing-remitting multiple sclerosis; SD = standard deviation; SDMT = Symbol Digit Modalities Test; sNfL = serum neurofilament light chain; SPMS = secondary progressive multiple sclerosis.

^a^Adjusted mean baseline sNfL values.

The detailed distribution of DMTs in both cohorts is provided in Supplementary Table 8. The follow-up assessments, including efficacy and safety outcomes, were conducted 90 days after baseline, during Q3, 2024. During the study period, no changes in DMTs occurred.

**Figure 2:**
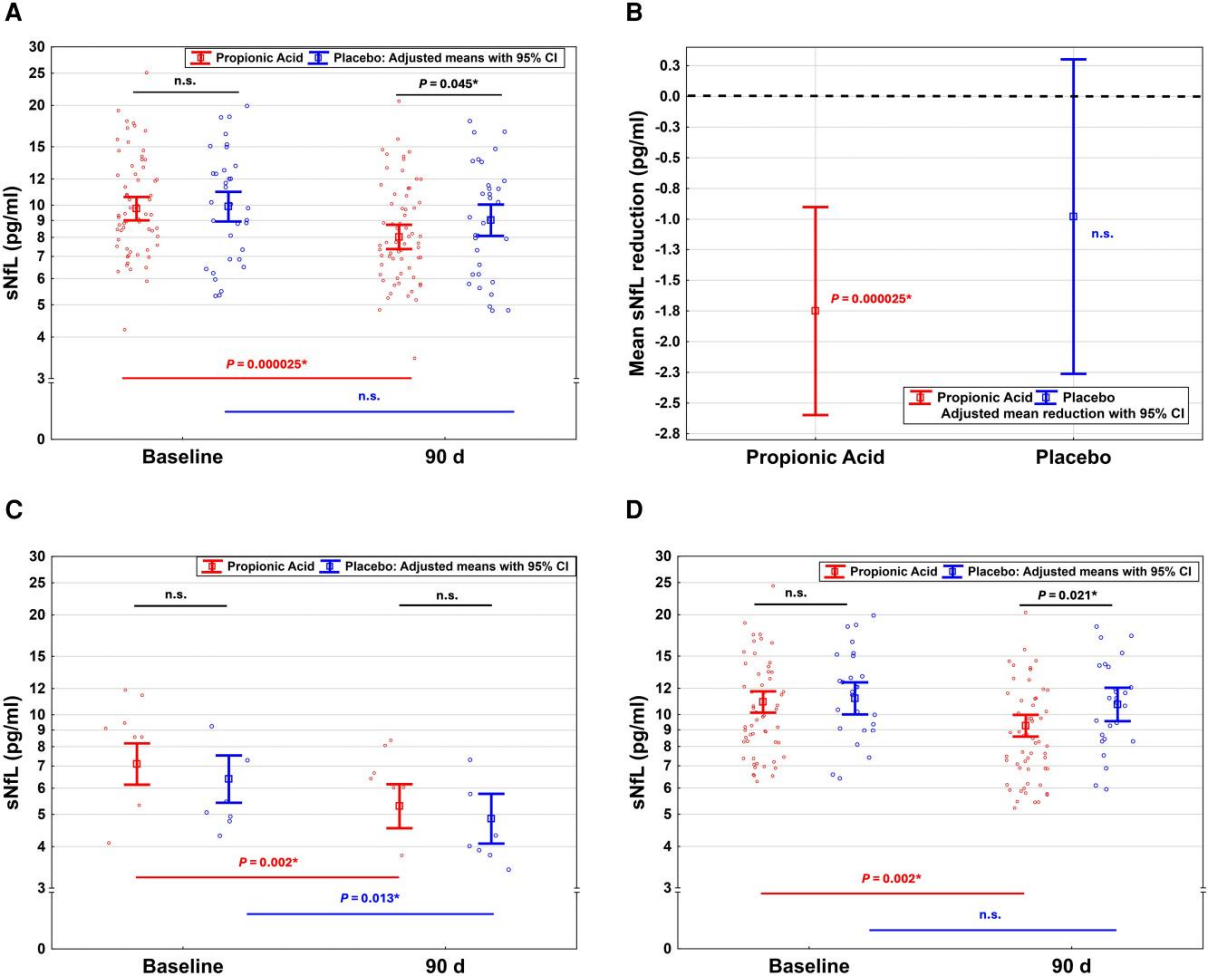
Effect of propionic acid supplementation on serum neurofilament light chain (sNfL) levels in multiple sclerosis. (A) A significant sNfL reduction was observed in the verum group (*P* = 0.000025), but not in the placebo group. At 90 days follow-up, mean sNfL levels were significantly lower in the propionic acid group than in the placebo group (*P* = 0.045). (B) Propionic acid supplementation led to a significant reduction in sNfL (−17.9%) compared to placebo over 90 days. (C) sNfL levels among participants with recent (within 12 months prior to baseline) disease-modifying therapy (DMT) change. Both subgroups exhibited significant reductions (verum: *P* = 0.002; placebo: *P* = 0.013), with no between-group differences. (D) Serum NfL changes in participants without recent DMT initiation (*n* = 84). A significant reduction was observed in the verum group (−15.2%; *P* = 0.002), but not in the placebo group (*P* = 0.60). At 90 days follow-up, sNfL levels were significantly lower in the verum group (*P* = 0.021). *Statistical significance according to a one-sided *P*-value. sNfL values above 30 pg/ml were excluded from visualization but included in statistical analyses. 90 d = 90 days of treatment; CI = confidence interval; n.s. not significant.

We observed a significant reduction in mean sNfL concentration in the verum group, but not in the placebo group (Fig. 2). The adjusted mean difference in sNfL levels between the PA and placebo groups at follow-up was 0.91 pg/ml (95% CI 0.02 to 1.79; *P* = 0.045). With PA supplementation, sNfL declined from 9.77 pg/ml (±8.81; 95% CI 9.00 to 10.60) at baseline to 8.02 pg/ml (±6.57; 95% CI 7.36 to 8.73) at follow-up (*P* = 0.000025), corresponding to a 17.9% reduction after 90 days (Fig. 2A and B). In the placebo group, no significant change was detected (baseline: 9.90 pg/ml ±5.8; 95% CI 8.94 to 10.98; follow-up: 8.92 pg/ml ±5.95; 95% CI 8.02 to 9.94; *P* = 0.14).

A new DMT had been initiated within 12 months prior to baseline in 13% (*n* = 9) of participants in the verum group and 24% (*n* = 8) of those in the placebo group. In these subgroups, sNfL levels decreased significantly in both treatment arms [PA: from 7.09 pg/ml (95% CI 6.14 to 8.18) to 5.29 pg/ml (95% CI 4.55 to 6.16); *P* = 0.002; placebo: from 6.39 pg/ml (95% CI 5.43 to 7.52) to 4.85 pg/ml (95% CI 4.08 to 5.77); *P* = 0.013; Fig. 2C], with no significant between-group differences.

Among participants without recent DMT initiation (*n* = 84), PA supplementation was associated with a significant decline in sNfL [from 10.92 pg/ml (95% CI 10.14 to 11.75) to 9.25 pg/ml (95% CI 8.57 to 9.98); *P* = 0.002], corresponding to a 15.2% reduction (Fig. 2D). In contrast, no effect was observed in the placebo group [from 11.20 pg/ml (95% CI 10.02 to 12.51) to 10.73 pg/ml (95% CI 9.56 to 12.04); *P* = 0.60]. While baseline sNfL did not differ between groups, including only individuals without recent DMT initiation (*P* = 0.70), levels were significantly lower in the verum group at follow-up (mean difference 1.48 pg/ml; 95% CI 0.28 to 2.68; *P* = 0.021).

A reduction in sNfL with PA supplementation was also observed among subgroups of participants receiving moderate-to-high efficacy DMTs (*P* = 0.0001, placebo *P* = 0.09, no significant between-group differences) and participants treated with anti-CD20 therapies (*P* = 0.0005, placebo *P* = 0.35). In individuals on anti-CD20 therapies, baseline sNfL values were higher in the PA group (10.2 pg/ml; 95% CI 9.19 to 11.33) than in the placebo arm (8.0 pg/ml, 95% CI 6.72 to 9.63; *P* = 0.01), and decreased to values comparable to placebo at follow-up [PA 8.0 pg/ml (95% CI 7.1 to 8.9) versus placebo 7.7 pg/ml (95% CI 6.33 to 9.27); *P* = 0.36].

Results from the secondary outcomes are presented in Table 2. As these analyses were not adjusted for multiplicity, the reported *P*-values should be interpreted accordingly, and future studies are recommended to confirm our results. There were no significant between-group effects for any secondary outcome. In the PA group, motor fatigue (FSMCmot) improved from 31.2 to 29.3 (*P* = 0.042), whereas no change was observed in the placebo group. Improvements in the cognitive fatigue subscore (FSMCcog) and in the total FSMC score did not reach statistical significance.

**Table 2:** Secondary outcomes

Outcomes		PA (*n* = 64)	Placebo (*n* = 32)
9-HPT; time, ss	BL	15.9 ± 6.4	17.8 ± 9.3
	FU	21.1 ± 10.7	22.4 ± 16.7
	*P*	**<0.001**	**<0.001**
10mWT; time, s	BL	3.9 ± 2.5	4.5 ± 3.2
	FU	4.0 ± 3.2	4.3 ± 3.2
	*P*	0.4	0.2
SDMT	BL	50.7 ± 16.9	51.7 ± 13.9
	FU	54.4 ± 16.2	55.5 ± 15.8
	*P*	**<0.001**	**0.0026**
FSMCcog	BL	28.6 ± 10.3	23.0 ± 9.9
	FU	26.5 ± 11.2	20.4 ± 9.1
	*P*	0.1	0.1
FSMCmot	BL	31.2 ± 9.9	24.9 ± 11.5
	FU	29.3 ± 10.7	24.4 ± 11.6
	*P*	**0.04**	0.7
FSMCtot	BL	59.4 ± 19.3	47.8 ± 21.0
	FU	55.8 ± 21.0	44.8 ± 19.7
	*P*	0.1	0.3
ESS	BL	7.7 ± 4.1	6.6 ± 4.7
	FU	7.6 ± 3.8	6.7 ± 4.3
	*P*	0.9	0.8
PCS (SF-36)	BL	56.7 ± 14.8	56.0 ± 16.2
	FU	58.6 ± 14.0	57.3 ± 16.4
	*P*	0.1	0.5
MCS (SF-36)	BL	51.1 ± 7.8	51.3 ± 7.2
	FU	50.2 ± 8.9	52.5 ± 7.4
	*P*	0.5	0.6

Data are presented as mean ± standard deviation; *P* indicates *P-*values; Bold values indicate statistical significance (*P* < 0.05). 10mWT = 10-Meter-Walking Test; 9-HPT = 9-Hole Peg Test; BL = baseline; ESS = Epworth Sleepiness Scale; FSMC = Fatigue Scale for Motor and Cognitive Functions; FU = follow-up; MCS (SF-36) = Mental Component Summary (Short Form 36 Health Survey); PA = propionic acid; PCS (SF-36) = Physical Component Summary (Short Form 36 Health Survey); SDMT = Symbol Digit Modalities Test.

We observed improvements in the SDMT and a deterioration in the 9HPT in both cohorts. Performance in the 10mWT remained unchanged in both treatment arms.

There were no intra- or intergroup effects in the ESS or in the MCS and PCS of the SF-36.

There were no significant changes in the vitamin D values from baseline (Q2, 2024) to follow-up (Q3, 2024), either in the verum (41 ng/ml ± 21 to 44 ng/ml ± 21) or in the placebo group (41 ng/ml ± 28 to 40 ng/ml ± 28).

Overall, 21 (31%) individuals from the PA group and 6 (18%) from the placebo arm reported side effects (*P* = 0.16; Table 3). The most frequent adverse events among the verum group were headache, bloating and stomach discomfort and among the placebo group, headache and diarrhoea. Bloating was significantly more frequent among the PA group (13% versus 0%; *P* = 0.026). No serious adverse events (SAEs) occurred. No clinically relevant changes in renal function parameters (creatinine and eGFR) were observed in either group during the study period. Two patients were hospitalized during the study, both deemed not related to the study: one individual had a lumbar spine surgery, and another participant (woman, 64-year-old, secondary progressive multiple sclerosis, placebo arm) had a clinical relapse with new and gadolinium-enhancing lesions in the MRI during the trial and was treated with steroids.

**Table 3:** Adverse events, safety population (*n* = 101)

Adverse events	PA (*n* = 67)	Placebo (*n* = 34)	*P*
Individuals experiencing at least one adverse event	21 (31%)	6 (18%)	0.16
Headache	9 (13%)	2 (6%)	0.32
Bloating	9 (13%)	–	0.026*
Stomach discomfort	5 (7%)	1 (3%)	0.66
Diarrhoea	3 (4%)	2 (6%)	1
Nausea	2 (3%)	–	0.55
Constipation	2 (3%)	–	0.55
Fatigue	1 (1%)	1 (3%)	1
Others (all transient):	3 (4%)	3 (9%)	0.4
Worsening of restless legs syndrome	–	1 (1%)	–
Decreased thirst	–	1 (1%)	–
Forearm pain	–	1 (1%)	–
Reduced appetite	1 (1%)	–	–
Visual disturbance, <24 h	1 (1%)	–	–
Delayed gastric emptying	1 (1%)	–	–

Data are presented as absolute numbers (*n*) and relative percentages (%); *statistically significant (Fisher’s Exact test).

In the PA group, three participants were lost during the study period. One 65-year-old woman with secondary progressive multiple sclerosis was diagnosed with metastatic carcinoma and withdrew from the study; one participant withdrew for family reasons; and one person was lost to follow-up.

In the placebo group, two individuals were lost during the study period. One 45-year-old man with relapsing-remitting multiple sclerosis reported paraesthesia and requested to withdraw from the trial; one participant was lost to follow-up.

## Discussion

The placebo-controlled MADAI trial provides evidence that PA supplementation mitigates neuroaxonal damage in people with multiple sclerosis, as indicated by significant decreases in sNfL levels. A within-group reduction in sNfL associated with add-on PA was also evident among participants receiving concomitant moderate-to-high efficacy DMTs, including those already on anti-CD20 agents. Assuming that high-efficacy DMTs effectively suppress inflammatory activity, the observed sNfL decline may point to a complementary neuroprotective or neuroregenerative potential of PA. The heterogeneity of the study cohort, including individuals with relapsing and progressive multiple sclerosis, spanning a broad age range and receiving diverse concomitant DMTs, enhances the generalizability of the findings and supports their applicability to real-world clinical practice. PA reduced sNfL levels by 17.9% after 90 days, an effect size comparable to that observed with ocrelizumab in the pivotal trials, where sNfL reductions of 20.7% in relapsing multiple sclerosis and decreases in plasma NfL of 12.4% in primary progressive multiple sclerosis were reported at 12 weeks.^18^ Approximately 80% of participants in our study had relapsing-remitting multiple sclerosis, and 20% had progressive forms of the disease. In the OPERA I and II (relapsing multiple sclerosis) and ORATORIO (primary progressive multiple sclerosis) trials, ocrelizumab-associated relative reductions in NfL were most pronounced within the first 12–24 weeks, with sustained effects observed up to 96 and 120 weeks, respectively.^18^ While the effect of PA after just 90 days indicates a rapid onset of action, it might be more evident with longer treatment periods, analogous with the persistent sNfL reductions under ocrelizumab.

To ensure that the observed decline in sNfL was not primarily attributable to recent initiation or changes in DMT, we conducted a prespecified subgroup analysis restricted to participants who had not modified their multiple sclerosis therapy within 12 months prior to baseline. In this population, PA supplementation remained associated with a statistically significant reduction in sNfL, supporting the robustness and internal validity of the primary findings. Moreover, follow-up sNfL levels were significantly lower in the verum group, further corroborating the treatment effect.

sNfL has emerged as a valuable biomarker for predicting both relapse risk and long-term disease progression in multiple sclerosis.^20-22^ Moreover, sNfL concentrations are associated with clinical outcomes such as PIRA and reaching EDSS milestones.^13,16,20,23^ Following treatment with ocrelizumab, persistently elevated sNfL levels remained predictive of long-term disability progression in both relapsing multiple sclerosis and primary progressive multiple sclerosis.^18^ These findings highlight the utility of sNfL in capturing both relapse-associated worsening (RAW) and smouldering non-relapsing disease activity, as seen in PIRA,^1^ and underscore the potential of PA to positively impact long-term disease trajectories in multiple sclerosis.

While most DMTs do not effectively reach the CNS,^4^ brain-penetrant PA may exert therapeutic effects both inside and outside the CNS.^10^ In the periphery, correction of PA deficiency shifts the immune balance by enhancing regulatory T cell function and modulating Th17 cells.^10^ These immunomodulatory capacities were accompanied by reduced relapse rates in an open-label analysis of 97 individuals with multiple sclerosis who received PA supplementation for at least one year.^10^ Within the CNS, PA and other SCFAs have demonstrated neuroprotective properties by modulating resident microglia, neurons and oligodendrocytes.^24-27^ Mechanistically, PA acts via free fatty acid receptors (FFAR) and histone deacetylase (HDACs) inhibition, processes implicated in axonal preservation, synaptic plasticity and antioxidant defence.^27-29^

In addition to the effects on the blood biomarker sNfL, PA supplementation was associated with a trend towards improvement in the motor subscore and overall score of the FSMC, findings that warrant confirmation in future studies. Fatigue is one of the most frequent and debilitating symptoms in multiple sclerosis.^30^ However, the underlying mechanisms remain poorly understood, and effective treatments are lacking. Although current evidence does not yet support a definitive role for enteric dysbiosis in the pathogenesis of fatigue symptoms, a small placebo-controlled trial reported that probiotic supplementation reduced anxiety in individuals with chronic fatigue after 2 months of treatment.^31^ Also, the association of sNfL with fatigue remains uncertain. Preliminary evidence from a small interventional trial reported parallel reductions in sNfL levels and fatigue severity following structured physical training.^32^ Further research is warranted to elucidate whether sNfL may serve as a surrogate marker of fatigue-related neuronal dysfunction and whether long-term supplementation with SCFAs could provide a therapeutic approach.

Three months of PA supplementation did not yield between-group effects in cognitive or physical test performance metrics. SDMT scores improved in both intervention and control groups, potentially reflecting a learning or practice effect. In contrast, performance on the 9HPT declined at follow-up assessment across all participants. This deterioration may be attributable to the extreme heat during the summer of 2024, which was recorded as the warmest on record in Europe.^33^ Elevated temperatures likely affected fine motor performance during the follow-up evaluations.

Diet is increasingly recognized as an environmental factor contributing to multiple sclerosis pathogenesis, and the motivation of people with multiple sclerosis to actively engage in lifestyle-based strategies for disease management is clear.^2,7,8^ Nevertheless, high-quality evidence supporting dietary interventions in multiple sclerosis remains limited, and their precise role and therapeutic potential have yet to be fully defined. Besides the MADAI data, a small pilot randomized trial of a modified Mediterranean diet (MMDI) reported a longitudinal decrease in serum NfL levels,^34^ but these findings require confirmation in larger, controlled studies. An adapted ketogenic diet has also been explored in a small study, where transient reductions in sNfL were observed.^17^ However, its clinical applicability is constrained by tolerability, dietary complexity and the potential risk of nutritional deficiencies. In contrast, PA supplementation is safe, well-tolerated and easy to integrate into daily life, offering a feasible and mechanistically grounded approach to modulating immune and neurodegenerative pathways in multiple sclerosis. Consistent with previous studies of up to 3 years of administration,^10^ we observed no serious adverse events related to PA intake. Mild bloating was significantly more common in the PA group than the placebo group, and we recommend taking PA with food to improve tolerability. As recently proposed in a holistic treatment approach to the management of multiple sclerosis,^1^ the MADAI study supports the use of PA supplementation as an add-on therapy.

This study is limited by the monocentric design, the 2:1 randomization, the short-term administration and the number of participants enrolled. While PA levels are decreased in multiple sclerosis,^10-12^ it was not possible to measure PA levels in participants’ blood, but we are working on a proposal and methodology to close this gap. It should be noted that serum NfL is not specific to CNS pathology and may be affected by other neurological or systemic conditions. Future research should examine the effects of long-term PA supplementation on clinical outcomes and whether, in combination with another SCFA, butyric acid (BA), the neuroprotective properties of PA are promoted.^27^ Moreover, the potential therapeutic use of PA across neurological disorders warrants further exploration, given evidence of its effects in peripheral nervous system cells.^35^

## Conclusion

This phase 2b MADAI trial provides evidence for the short-term effectiveness and safety of PA supplementation in reducing sNfL, a biomarker for neuroaxonal loss, in multiple sclerosis. PA emerges as a promising supplement to address the unmet need for agents that modulate both peripheral and CNS-resident immune responses while promoting neuroprotection. Therefore, it should be considered an add-on approach for people with multiple sclerosis. The MADAI trial lays the groundwork for future long-term, phase 3 clinical studies.

## Supplementary Material

awag099_Supplementary_Data

## Data Availability

The de-identified individual participant data that underlie the results reported in this article, along with the study protocol and statistical analysis plan, will be made available upon reasonable request to the corresponding author. Requests will be reviewed by the study steering committee and may require a data-sharing agreement. Data will be shared for academic, non-commercial research purposes only, in accordance with ethical approvals and participant consent.
