# Supplementary material for: Propionic acid in multiple sclerosis: a phase 2b, double-blind, randomized placebo-controlled trial
Source: Brain. 2026 Jul 6;149(7):2286–94. doi: 10.1093/brain/awag099 (PMC13337218; doi:10.1093/brain/awag099)
Supplement: awag099_Supplementary_Data [file awag099_supplementary_data.pdf]

Supplementary Material

Article Title: Propionic acid in multiple sclerosis: a phase 2b, double-blind, randomised placebo-controlled trial

Authors: Tobias Moser Prof[1], Wolfgang Hitzl[2,3], Tiago Lerda-Casaccia MD[1], Michael Unterhofer MD[1], Rina Demjaha[4], Maria Martinez-Serrat[4], Michael Khalil Prof[4], Andrea Harrer[1,5], Belinda Böhm MSc[6], Peter Hofbauer[7], Janne Cadamuro Prof[8], Ursula Huber-Schönauer MD[9], Eugen Trinka MD, MSc, FRCP[1,10] and Peter Wipfler Prof[1]

Author affiliations:

1. Department of Neurology, Christian Doppler University Hospital, Paracelsus Medical University and Center for Cognitive Neuroscience, European Reference Network EpiCARE, 5020, Salzburg, Austria
2. Paracelsus Medical University Salzburg, Department of Ophthalmology and Optometry, Salzburg, Austria.
3. Research Program Experimental Ophthalmology & Glaucoma Research, Paracelsus Medical University Salzburg, Austria
4. Department of Neurology, Medical University of Graz, 8036, Graz, Austria
5. Department of Dermatology and Allergology, University Hospital Salzburg, Paracelsus Medical University, 5020, Salzburg, Austria
6. Department for Health Sciences, Physiotherapy, Salzburg University of Applied Sciences, 5412, Puch/Salzburg, Austria;
7. Landesapotheke Salzburg, Betrieb des Landes Salzburg, 5020, Salzburg, Austria
8. Department of Laboratory Medicine, Paracelsus Medical University, 5020, Salzburg, Austria;
9. Department of Nuclear Medicine and Endocrinology, University Hospital Salzburg, Paracelsus Medical University, 5020, Salzburg, Austria
10. Neuroscience Institute, Christian Doppler University Hospital, Paracelsus Medical University and Center for Cognitive Neuroscience, 5020, Salzburg, Austria

Supplementary Methods

Procedures

Routine laboratory analyses were conducted at the Department of Laboratory Medicine, University Hospital Salzburg. Clinical chemistry parameters were analysed on the COBAS8100 platform (Roche Diagnostics, Rotkreuz, Switzerland), using the according assay from the same company. The tests served to exclude relevant comorbid conditions prior to

inclusion and to monitor for potential adverse effects during the intervention. All assays were carried out according to the manufacturers' instructions, internal quality controls (IQC) were measured twice daily at different concentrations and external quality controls (EQC) were performed every 3 months. The analysis of vitamin D was carried out in the hormone laboratory of the Department for Nuclear Medicine and Endocrinology from the University Hospital Salzburg, Austria. 25-Monohydroxy-vitamin D was measured in serum samples within 3 hours of blood collection. Analyses were performed on the Abbott Alinity i analyser using the Abbott 25OH Vitamin D Reagent Kit (08P4522) as well as 25OH Vitamin D Calibrators (08P4501). Internal quality assurance was performed with the Technopath quality assurance material Multichem IA plus (08P86-10), while external quality assurance was carried out by the *Referenzinstitut für Bioanalytik in Bonn*, Germany. Both laboratories are certified according to ISO 9001: 2015 and work in accordance with the accreditation standard for medical laboratories ISO 15189: 2022.

Cognitive performance was assessed using the Symbol Digit Modalities Test (SDMT), a validated tool for evaluating cognitive processing speed in individuals with multiple sclerosis. In this test, participants are required to match abstract symbols to corresponding numbers using a reference key. The score is based on the number of correct symbol-digit pairings completed within 90 seconds, with higher scores indicating better cognitive function.

Physical performance was evaluated using the Nine-Hole Peg Test (9HPT) and the 10-Meter Walk Test (10mWT). The 9HPT is a standardized and widely accepted measure of manual dexterity and fine motor coordination of the upper limbs. Participants are instructed to place nine pegs into holes on a board and then remove them, one at a time, using one hand. Data displayed accounts for the fastest performance in the 9HPT. The procedure is repeated two times per hand to ensure reliability. Faster completion times indicate better manual dexterity. The 10mWT was used to assess lower limb function by measuring walking speed. Participants walked a straight, flat 10-meter course at their maximum pace. Timing was limited to the middle 6 meters - between the 2- and 8-meter marks - to exclude acceleration and deceleration phases.

Participants received a structured online questionnaire via email at baseline and follow-up. The questionnaire included validated instruments to assess quality of life, fatigue, and daytime sleepiness. Quality of life was measured using the 36-Item Short Form Health Survey questionnaire (SF-36), a generic and widely used instrument consisting of eight scales: physical functioning (PF), role physical (RP), bodily pain (BP), general health (GH), vitality (VT), social

functioning (SF), role emotional (RE), and mental health (MH). The SF-36 provides two summary scores: the Physical Component Summary (PCS) and the Mental Component Summary (MCS), both of which are calculated using a specific algorithm that combines the scales mentioned above reflecting overall physical and mental health status, respectively. Higher scores indicate better health-related quality of life in the corresponding dimensions. Each domain is scored separately and transformed to a scale from 0 (poor health) to 100 (optimal health). Scores are interpreted as follows: excellent (>60), above average (51-60, average to slightly below (41-50), moderately below average (31-40) and significant impairment (<30). The SF-36 is considered the gold-standard that measures the perceived health status and is commonly used for multiple sclerosis. Fatigue was measured with the Fatigue Scale for Motor and Cognitive Functions (FSMC), a 20-item questionnaire that separately assesses motor (FSMCmot) and cognitive (FSMCcog) aspects of fatigue. The total score (FSMCtot) ranges from 20 to 100, with fatigue severity classified as mild (≥43), moderate (≥53), and severe (≥63). Daytime sleepiness was evaluated using the Epworth Sleepiness Scale (ESS), a validated, self-administered questionnaire consisting of 8 items rated on a 4-point scale (0–3). The ESS assesses the likelihood of dozing off in various daily situations, yielding a total score between 0 and 24. Higher scores generally indicate more pronounced daytime sleepiness. A score above 10 suggests mild, above 13 moderate and above 16 severe excessive daytime sleepiness. The questionnaire was administered using EvaSys (21337 Lüneburg, Germany) and responses were collected in .csv format and processed in Microsoft Excel.

Supplementary Statistics and Results

Covariates included in the GEE models were fixed at the following values: age = 47.1 years, BMI = 25.5 kg/m², creatinine = 0.81 mg/dL, and sNfL = 12.6 pg/mL (Supplementary Table 1).

Detailed parameter estimates are presented in Supplementary Tables 2–6 for the full cohort and relevant subgroups, including participants with and without moderate-to-high efficacy DMTs, and with or without anti-CD20 treatment.

Raw sNfL data of the two participants excluded from primary analysis:
PA group - baseline: 6.4 pg/mL; 90-day follow-up: 19.3 pg/mL
Placebo group - baseline: 25.8 pg/mL; 90-day follow-up: 12.1 pg/mL

Supplementary Figure 1. Histogram of sNfL values with corresponding estimated probabilities illustrating the deviation from normality

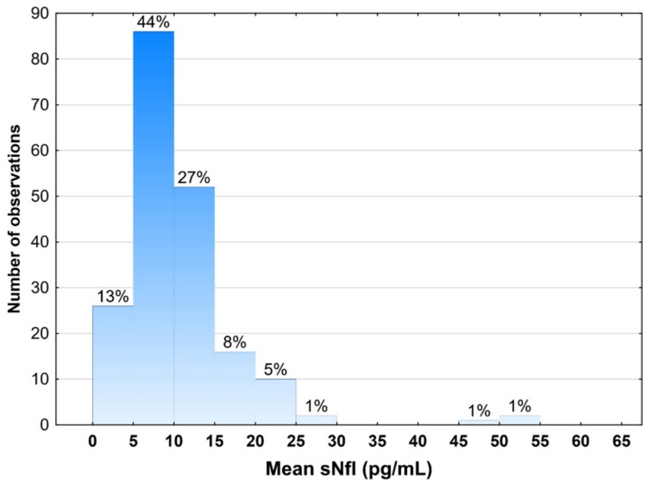

Supplementary Figure 2. Density plot of a Tweedie distribution (1 < φ < 2) representing a Poisson–gamma compound distribution, included to visualize the modelled right-skewed distribution of sNfL values

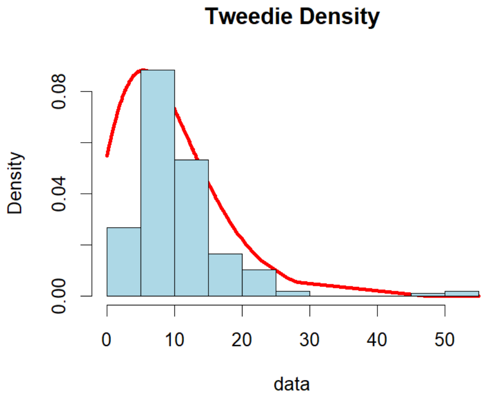

Supplementary Tables

Supplementary Table 1 Descriptive statistics of the continuously distributed covariates

		Minimum	Maximum	Mean	Std. Deviation
Covariate	age	25.00	70.00	47.1	11.96
	BMI	17.26	54.33	25.5	6.62
	Creatinine (baseline)	0.390	1.360	0.81	0.17

sNfL (baseline)	2.89	54.76	12.6	7.86

age = age in years; BMI = body mass index (kg/m²); Creatinine (baseline) = serum creatinine at baseline (mg/dL); sNfL (baseline) = serum neurofilament light chain at baseline (pg/mL); Minimum = minimum value; Maximum = maximum value; Mean = mean value; Std. Deviation = standard deviation.

Supplementary Table 2 (A) Parameter estimates including coefficients, standard errors, test statistics, and p-values; and (B) planned contrasts of adjusted mean serum neurofilament light chain (sNfL) values (primary outcome) between the propionic acid (Verum) and placebo groups, as well as over time (baseline and 90-day follow-up) - across the whole cohort

Verum group: baseline n =67, follow-up (90 days) n =63;
Placebo group: baseline n =34, follow-up (90 days) n =31.

(A)

Parameter	B	Std. Error	95% Wald Confidence Interval		Hypothesis Test		
			Lower	Upper	Wald Chi-Square	df	Sig.
(Intercept)	1.08	0.15	0.79	1.37	54.42	1.00	<0.001
[baseline]	0.20	0.05	0.10	0.29	16.59	1.00	<0.001
[90 days follow-up]	0[a]	-	-	-	-	-	-
[Group=Placebo]	0.12	0.06	-0.01	0.24	3.46	1.00	0.063
[Group=Verum]	0[a]	-	-	-	-	-	-
[Group=Placebo]*[baseline]	-0.10	0.09	-0.27	0.06	1.45	1.00	0.229
[Group=Placebo]*[90 days follow-up]	0[a]	-	-	-	-	-	-
[Group=Verum]*[baseline]	0[a]	-	-	-	-	-	-
[Group=Verum]*[90 days follow-up]	0[a]	-	-	-	-	-	-
age	0.01	0.00	0.01	0.02	42.35	1.00	<0.001
BMI	-0.01	0.00	-0.02	0.00	7.70	1.00	0.006
Creatinine (baseline)	0.16	0.12	-0.08	0.40	1.70	1.00	0.192
sNfL (baseline)	0.04	0.00	0.03	0.04	280.22	1.00	<0.001
(Scale)	0.24	-	-	-	-	-	-

B = regression coefficient; Std. Error = standard error; 95% Wald CI = 95% Wald confidence interval; Wald Chi-Square = Wald chi-square test statistic; df = degrees of freedom; Sig. = significance (p-value); [Group=Placebo] = placebo group; [Group=Verum] = propionic acid group. [a] Set to zero because this parameter is redundant.

(B)

Pairwise Comparisons

(I) time*Group		Mean Difference (I-J)	Std. Error	df	Sig.	95% Wald Confidence Interval for Difference	
						Lower	Upper
[baseline]*[Group=Placebo]	[baseline]*[Group=Verum]	0.14	0.58	1	0.41	-0.81	1.09
	[90 days follow-up]*[Group=Placebo]	0.98	0.65	1	0.14	-0.10	2.06
	[90 days follow-up]*[Group=Verum]	1.89[a]	0.56	1	<0.001	0.98	2.80
[baseline]*[Group=Verum]	[baseline]*[Group=Placebo]	0.14	0.58	1	0.41	-1.09	0.81
	[90 days follow-up]*[Group=Placebo]	0.84	0.56	1	0.07	-0.08	1.77
	[90 days follow-up]*[Group=Verum]	1.75[a]	0.43	1	<0.001	1.04	2.46
[90 days follow-up]*[Group=Placebo]	[baseline]*[Group=Placebo]	0.98	0.65	1	0.14	-2.06	0.10
	[baseline]*[Group=Verum]	0.84	0.56	1	0.07	-1.77	0.08
	[90 days follow-up]*[Group=Verum]	0.91[a]	0.54	1	0.045	0.02	1.79
[90 days follow-up]*[Group=Verum]	[baseline]*[Group=Placebo]	-1.89[a]	0.56	1	<0.001	-2.80	-0.98
	[baseline]*[Group=Verum]	-1.75[a]	0.43	1	<0.001	-2.46	-1.04
	[90 days follow-up]*[Group=Placebo]	-0.91[a]	0.54	1	0.045	-1.79	-0.02

Pairwise comparisons of estimated marginal means based on the original scale of dependent variable sNfL. Std. Error = standard error; df = degrees of freedom; Sig. = significance (p-value); [Group=Placebo] = placebo group; [Group=Verum] = propionic acid group. [a] Mean difference is significant at the 0.05 level.

Supplementary Table 3 (A) Parameter estimates including coefficients, standard errors, test statistics, and p-values; and (B) planned contrasts of adjusted mean serum neurofilament light chain (sNfL) values (primary outcome) between the propionic acid (Verum) and placebo groups, as well as over time (baseline and 90-day follow-up) - for participants not receiving moderate-to-high efficacy disease-modifying therapies

Verum group: baseline $n = 26$, follow-up (90 days) $n = 23$;
Placebo group: baseline $n = 16$, follow-up (90 days) $n = 15$.

(A)

Parameter	B	Std. Error	95% Wald Confidence Interval		Hypothesis Test		
			Lower	Upper	Wald Chi-Square	df	Sig.
(Intercept)	1.171	0.2194	0.741	1.602	28.501	1	<0.001
[baseline]	0.155	0.0743	0.009	0.300	4.339	1	0.037
[90 days follow-up]	0[a]	-	-	-	-	-	-
[Group=Placebo]	0.180	0.0884	0.007	0.353	4.163	1	0.041
[Group=Verum]	0[a]	-	-	-	-	-	-

[baseline] * [Group=Placebo]	-0.108	0.1200	-0.343	0.127	0.815	1	0.367
[baseline] * [Group=Verum]	0[a]	-	-	-	-	-	-
[90 days follow-up] * [Group=Placebo]	0[a]	-	-	-	-	-	-
[90 days follow-up] * [Group=Verum]	0[a]	-	-	-	-	-	-
age	0.009	0.0034	0.002	0.015	6.415	1	0.011
BMI	-0.004	0.0056	-0.015	0.006	0.647	1	0.421
Creatinine (baseline)	0.303	0.1723	-0.034	0.641	3.101	1	0.078
sNfL (baseline)	0.043	0.0034	0.036	0.049	155.339	1	<0.001
(Scale)	0.235	-	-	-	-	-	-

B = regression coefficient; Std. Error = standard error; 95% Wald CI = 95% Wald confidence interval; Wald Chi-Square = Wald chi-square test statistic; df = degrees of freedom; Sig. = significance (p-value); [Group=Placebo] = placebo group; [Group=Verum] = propionic acid group. [a] Set to zero because this parameter is redundant.

(B)

Pairwise Comparisons

(I) time*Group		Mean Difference (I-J)	Std. Error	df	Sig.	95% Wald Confidence Interval for Difference	
						Lower	Upper
[baseline]*[Group=Placebo]	[baseline]*[Group=Verum]	0.86	1.00	1	0.39	-1.11	2.83
	[90 days follow-up]*[Group=Placebo]	0.56	1.14	1	0.62	-1.67	2.80
	[90 days follow-up]*[Group=Verum]	2.51[a]	0.98	1	0.01	0.59	4.43
[baseline]*[Group=Verum]	[baseline]*[Group=Placebo]	-0.86	1.00	1	0.39	-2.83	1.11
	[90 days follow-up]*[Group=Placebo]	-0.30	1.00	1	0.77	-2.27	1.67
	[90 days follow-up]*[Group=Verum]	1.65[a]	0.79	1	0.04	0.10	3.21
[90 days follow-up]*[Group=Placebo]	[baseline]*[Group=Placebo]	-0.56	1.14	1	0.62	-2.80	1.67
	[baseline]*[Group=Verum]	0.30	1.00	1	0.77	-1.67	2.27
	[90 days follow-up]*[Group=Verum]	1.95[a]	0.98	1	<0.05	0.03	3.87
[90 days follow-up]*[Group=Verum]	[baseline]*[Group=Placebo]	-2.51[a]	0.98	1	0.01	-4.43	-0.59

	[baseline]*[Group=Verum]	-1.65[a]	0.79	1	0.04	-3.21	-0.10
	[90 days follow-up]*[Group=Placebo]	-1.95[a]	0.98	1	<0.05	-3.87	-0.03

Pairwise comparisons of estimated marginal means based on the original scale of dependent variable sNfL. Std. Error = standard error; df = degrees of freedom; Sig. = significance (p-value); [Group=Placebo] = placebo group; [Group=Verum] = propionic acid group. [a] Mean difference is significant at the 0.05 level.

Supplementary Table 4 (A) Parameter estimates including coefficients, standard errors, test statistics, and p-values; and (B) planned comparisons of adjusted mean serum neurofilament light chain (sNfL) values (primary outcome) between the propionic acid (Verum) and placebo groups, as well as over time (baseline and 90-day follow-up) - for participants receiving moderate-to-high efficacy disease-modifying therapies

Verum group: baseline n = 41, follow-up (90 days) n = 40;
Placebo group: baseline n = 18, follow-up (90 days) n = 16.

(A)

Parameter Estimates

Parameter	B	Std. Error	95% Wald Confidence Interval		Hypothesis Test		
			Lower	Upper	Wald Chi-Square	df	Sig.
(Intercept)	1.244	0.1905	0.871	1.618	42.638	1	<0.001
[baseline]	0.232	0.0623	0.110	0.354	13.845	1	<0.001
[90 days follow-up]	0[a]	-	-	-	-	-	-
[Group=Placebo]	-0.011	0.0866	-0.180	0.159	0.015	1	0.902
[Group=Verum]	0[a]	-	-	-	-	-	-
[baseline] * [Group=Placebo]	-0.060	0.1176	-0.290	0.171	0.259	1	0.611
[baseline] * [Group=Verum]	0[a]	-	-	-	-	-	-
[90 days follow-up] * [Group=Placebo]	0[a]	-	-	-	-	-	-
[90 days follow-up] * [Group=Verum]	0[a]	-	-	-	-	-	-
age	0.016	0.0024	0.011	0.021	44.919	1	<0.001
BMI	-0.013	0.0040	-0.021	-0.005	11.012	1	0.001

Creatinine (baseline)	0.105	0.1742	-0.236	0.447	0.366	1	0.545
sNfL (baseline)	0.035	0.0030	0.029	0.041	140.390	1	<0.001
(Scale)	0.232	-	-	-	-	-	-

B = regression coefficient; Std. Error = standard error; 95% Wald CI = 95% Wald confidence interval; Wald Chi-Square = Wald chi-square test statistic; df = degrees of freedom; Sig. = significance (p-value); [Group=Placebo] = placebo group; [Group=Verum] = propionic acid group. [a] Set to zero because this parameter is redundant.

(B)

Pairwise Comparisons

(I) time*Group		Mean Difference (I-J)	Std. Error	df	Sig.	95% Wald Confidence Interval for Difference	
						Lower	Upper
[baseline]*[Group=Placebo]	[baseline]*[Group=Verum]	-0.66	0.74	1	0.38	-2.10	0.79
	[90 days follow-up]*[Group=Placebo]	1.42	0.82	1	0.09	-0.19	3.03
	[90 days follow-up]*[Group=Verum]	1.34	0.71	1	0.06	-0.05	2.72
[baseline]*[Group=Verum]	[baseline]*[Group=Placebo]	0.66	0.74	1	0.38	-0.79	2.10
	[90 days follow-up]*[Group=Placebo]	2.07[a]	0.69	1	0.003	0.72	3.43
	[90 days follow-up]*[Group=Verum]	1.99[a]	0.54	1	<0.001	0.94	3.04
[90 days follow-up]*[Group=Placebo]	[baseline]*[Group=Placebo]	-1.42	0.82	1	0.085	-3.03	0.19
	[baseline]*[Group=Verum]	-2.07[a]	0.69	1	0.003	-3.43	-0.72
	[90 days follow-up]*[Group=Verum]	-0.08	0.66	1	0.90	-1.37	1.20
[90 days follow-up]*[Group=Verum]	[baseline]*[Group=Placebo]	-1.34	0.71	1	0.06	-2.72	0.05
	[baseline]*[Group=Verum]	-1.99[a]	0.54	1	<0.001	-3.04	-0.94

[90 days follow-up]*[Group=Placebo]	0.08	0.66	1	0.90	-1.20	1.37

Pairwise comparisons of estimated marginal means based on the original scale of dependent variable sNfL. Std. Error = standard error; df = degrees of freedom; Sig. = significance (p-value); [Group=Placebo] = placebo group; [Group=Verum] = propionic acid group. [a] Mean difference is significant at the 0.05 level.

Supplementary Table 5 (A) Parameter estimates including coefficients, standard errors, test statistics, and p-values; and (B) planned contrasts of adjusted mean serum neurofilament light chain (sNfL) values (primary outcome) between the propionic acid (Verum) and placebo groups, as well as over time (baseline and 90-day follow-up) - for participants without anti-CD20 treatment

Verum group: baseline *n* = 40, follow-up (90 days) *n* = 37;
Placebo group: baseline *n* = 22, follow-up (90 days) *n* = 21.

(A)

Parameter	B	Std. Error	95% Wald Confidence Interval		Hypothesis Test		
			Lower	Upper	Wald Chi-Square	df	Sig.
(Intercept)	1.190	0.1784	0.840	1.539	44.457	1	<0.001
[baseline]	0.175	0.0598	0.058	0.293	8.586	1	0.003
[90 days follow-up]	0[a]	-	-	-	-	-	-
[Group=Placebo]	0.156	0.0734	0.013	0.300	4.541	1	0.033
[Group=Verum]	0[a]	-	-	-	-	-	-
[baseline] * [Group=Placebo]	-0.071	0.0998	-0.266	0.125	0.500	1	0.479
[baseline] * [Group=Verum]	0[a]	-	-	-	-	-	-
[90 days follow-up] * [Group=Placebo]	0[a]	-	-	-	-	-	-
[90 days follow-up] * [Group=Verum]	0[a]	-	-	-	-	-	-
age	0.010	0.0023	0.005	0.015	18.222	1	<0.001
BMI	-0.009	0.0048	-0.018	<0.001	3.480	1	0.062
Creatinine (baseline)	0.242	0.1377	-0.028	0.512	3.082	1	0.079
sNfL (baseline)	0.045	0.0029	0.040	0.051	237.209	1	<0.001
(Scale)	0.218	-	-	-	-	-	-

B = regression coefficient; Std. Error = standard error; 95% Wald CI = 95% Wald confidence interval; Wald Chi-Square = Wald chi-square test statistic; df = degrees of freedom; Sig. = significance (p-value);

[Group=Placebo] = placebo group; [Group=Verum] = propionic acid group. [a] Set to zero because this parameter is redundant.

(B)

Pairwise Comparisons

(I) time*Group		Mean Difference (I-J)	Std. Error	df	Sig.	95% Wald Confidence Interval for Difference	
						Lower	Upper
[baseline]*[Group=Placebo]	[baseline]*[Group=Verum]	0.94	0.76	1	0.22	-0.56	2.43
	[90 days follow-up]*[Group=Placebo]	1.13	0.86	1	0.19	-0.56	2.82
	[90 days follow-up]*[Group=Verum]	2.62[a]	0.74	1	<0.001	1.16	4.07
[baseline]*[Group=Verum]	[baseline]*[Group=Placebo]	-0.94	0.76	1	0.22	-2.43	0.56
	[90 days follow-up]*[Group=Placebo]	0.20	0.73	1	0.79	-1.24	1.64
	[90 days follow-up]*[Group=Verum]	1.68[a]	0.57	1	0.003	0.56	2.80
[90 days follow-up]*[Group=Placebo]	[baseline]*[Group=Placebo]	-1.13	0.86	1	0.19	-2.82	0.56
	[baseline]*[Group=Verum]	-0.20	0.73	1	0.79	-1.64	1.24
	[90 days follow-up]*[Group=Verum]	1.48[a]	0.71	1	0.04	0.08	2.88
[90 days follow-up]*[Group=Verum]	[baseline]*[Group=Placebo]	-2.62[a]	0.74	1	<0.001	-4.07	-1.16
	[baseline]*[Group=Verum]	-1.68[a]	0.57	1	0.003	-2.80	-0.56
	[90 days follow-up]*[Group=Placebo]	-1.48[a]	0.71	1	0.04	-2.88	-0.08

Pairwise comparisons of estimated marginal means based on the original scale of dependent variable sNfL. Std. Error = standard error; df = degrees of freedom; Sig. = significance (p-value); [Group=Placebo] = placebo group; [Group=Verum] = propionic acid group. [a] Mean difference is significant at the 0.05 level.

Supplementary Table 6 (A) Parameter estimates including coefficients, standard errors, test statistics, and p-values; and (B) planned contrasts of adjusted mean serum neurofilament light chain (sNfL) values (primary outcome) between the propionic acid

(Verum) and placebo groups, as well as over time (baseline and 90-day follow-up) - for participants with anti-CD20 treatment

Verum group: baseline $n = 27$, follow-up (90 days) $n = 26$;
Placebo group: baseline $n = 12$, follow-up (90 days) $n = 10$.

(A)

Parameter Estimates

Parameter	B	Std. Error	95% Wald Confidence Interval		Hypothesis Test		
			Lower	Upper	Wald Chi-Square	df	Sig.
(Intercept)	1.303	0.2383	0.836	1.770	29.897	1	<0.001
[baseline]	0.247	0.0753	0.099	0.394	10.746	1	0.001
[90 days follow-up]	0a	-	-	-	-	-	-
[Group=Placebo]	-0.040	0.1142	-0.264	0.184	0.125	1	0.724
[Group=Verum]	0a	-	-	-	-	-	-
[baseline] * [Group=Placebo]	-0.198	0.1512	-0.494	0.098	1.712	1	0.191
[baseline] * [Group=Verum]	0a	-	-	-	-	-	-
[90 days follow-up] * [Group=Placebo]	0a	-	-	-	-	-	-
[90 days follow-up] * [Group=Verum]	0a	-	-	-	-	-	-
age	0.020	0.0031	0.014	0.026	40.822	1	<0.001
BMI	-0.012	0.0044	-0.020	-0.003	7.144	1	0.008
Creatinine (baseline)	-0.076	0.2341	-0.535	0.383	0.104	1	0.747
sNfL (baseline)	0.029	0.0034	0.022	0.035	69.410	1	<0.001
(Scale)	0.235	-	-	-	-	-	-

B = regression coefficient; Std. Error = standard error; 95% Wald CI = 95% Wald confidence interval; Wald Chi-Square = Wald chi-square test statistic; df = degrees of freedom; Sig. = significance (p-value); [Group=Placebo] = placebo group; [Group=Verum] = propionic acid group. [a] Set to zero because this parameter is redundant.

(B)

Pairwise Comparisons

(I) time*Group		Mean Difference (I-J)	Std. Error	df	Sig.	95% Wald Confidence Interval for Difference	
						Lower	Upper
[baseline]*[Group=Placebo]	[baseline]*[Group=Verum]	-2.16[a]	0.93	1	0.01	-3.99	-0.33

	[90 days follow-up]*[Group=Placebo]	0.38	1.03	1	0.35	-1.63	2.40
	[90 days follow-up]*[Group=Verum]	0.07	0.88	1	0.47	-1.65	1.79
[baseline]*[Group=Verum]	[baseline]*[Group=Placebo]	-2.16[a]	0.93	1	0.01	0.33	3.99
	[90 days follow-up]*[Group=Placebo]	2.55[a]	0.94	1	0.01	0.71	4.39
	[90 days follow-up]*[Group=Verum]	-2.23[a]	0.68	1	<0.001	0.89	3.57
[90 days follow-up]*[Group=Placebo]	[baseline]*[Group=Placebo]	-0.38	1.03	1	0.35	-2.40	1.63
	[baseline]*[Group=Verum]	2.55[a]	0.94	1	0.01	-4.39	-0.71
	[90 days follow-up]*[Group=Verum]	-0.32	0.88	1	0.36	-2.05	1.42
[90 days follow-up]*[Group=Verum]	[baseline]*[Group=Placebo]	-0.07	0.88	1	0.47	-1.79	1.65
	[baseline]*[Group=Verum]	-2.23[a]	0.68	1	<0.001	-3.57	-0.89
	[90 days follow-up]*[Group=Placebo]	0.32	0.88	1	0.36	-1.42	2.05

Pairwise comparisons of estimated marginal means based on the original scale of dependent variable sNfL. Std. Error = standard error; df = degrees of freedom; Sig. = significance (p-value); [Group=Placebo] = placebo group; [Group=Verum] = propionic acid group. [a] Mean difference is significant at the 0.05 level.

Supplementary Table 7 Overview of missing data for secondary outcomes

**Outcomes:**		**PA (*n* = 67)**	**Placebo (*n* = 34)**
**9HPT [s]**	BL	0 (0%)	1 (3%)
	FU	3 (4%)	3 (9%)
**10mWT [s]**	BL	1 (1%)	2 (6%)
	FU	4 (6%)	5 (15%)
**SDMT**	BL	0 (0%)	2 (6%)
	FU	3 (4%)	3 (9%)
**FSMCcog**	BL	2 (3%)	4 (12%)
	FU	4 (6%)	3 (9%)
**FSMCmot**	BL	3 (4%)	4 (12%)
	FU	4 (6%)	3 (9%)
**FSMCtot**	BL	2 (3%)	3 (9%)
	FU	4 (6%)	3 (9%)
**ESS**	BL	3 (4%)	2 (6%)
	FU	4 (6%)	2 (6%)
**PCS (SF-36)**	BL	2 (3%)	4 (12%)
	FU	3 (4%)	4 (12%)
**MCS (SF-36)**	BL	5 (7%)	3 (9%)
	FU	5 (7%)	3 (9%)

PA = Propionic acid; BL = Baseline; FU = Follow-up; 9HPT = 9-Hole Peg Test; 10mWT = 10-Meter-Walking Test; SDMT = Symbol Digit Modalities Test; FSMC = Fatigue Scale for Motor and Cognitive Functions; ESS = Epworth Sleepiness Scale; PCS (SF-36) = Physical Component Summary (Short Form 36 Health Survey); MCS (SF-36) = Mental Component Summary (Short Form 36 Health Survey).

Supplementary Table 8 Disease-modifying therapy (DMT) use in the propionic acid (PA) and placebo groups

DMT category	PA<br>(*n* = 67)	Placebo<br>(*n* = 34)
**Glatiramer acetate**	2 (3%)	1 (3%)
**Interferons**	2 (3%)	1 (3%)
**Dimethyl fumarate**	4 (6%)	6 (18%)
**Teriflunomide**	4 (6%)	0 (0%)
**Cladribine**	2 (3%)	1 (3%)
**Alemtuzumab**	0 (0%)	1 (3%)
**S1P receptor modulators**	8 (12%)	4 (12%)
**Anti-CD20 antibodies**	27 (40%)	12 (35%)
**Natalizumab**	4 (6%)	0 (0%)
**Azathioprine**	0 (0%)	1 (3%)
**No DMT**	13 (19%)	5 (15%)
**Unknown**	1 (1%)	1 (3%)
